# Second and Third Virial Coefficients for Hydrogen

**DOI:** 10.6028/jres.068A.011

**Published:** 1964-02-01

**Authors:** R. D. Goodwin, D. E. Diller, H. M. Roder, L. A. Weber

**Affiliations:** Cryogenic Engineering Laboratory, National Bureau of Standards, Boulder, Colo.

## Abstract

Second and third virial coefficients for parahydrogen have been derived from closely spaced PVT data from 24 to 100 °K. They are in good agreement at 100 °K with published data for normal hydrogen. Analytical representations of the combined data from about 20 to 423 °K are presented which may be useful in computation of thermodynamic functions of the gas. These formulas are related to those resulting from the use of the Lennard-Jones potential.

## 1. Introduction

As part of an extensive program for determining the thermodynamic and transport properties of fluid parahydrogen, we have recently published new data on the P-V-T surface from 15 to 100 °K [1].^1^ Earlier, we gave preliminary values of the second and third virial coefficients, *B*(*T*) and *C*(*T*), in a paper describing the apparatus [2].

In this paper we present final values of the virial coefficients derived from the smoothed compressibility data [1]. These differ but slightly from the preliminary values. Also we present and examine analytical representations of *B*(*T*) and *C*(*T*) which may be useful for computing thermodynamic functions of the gas.

In a forthcoming paper [3] we have used the following arbitrary representations [4] to fit our closely spaced data in order to facilitate computations of thermofunctions below 100 °K:

$$B=\sum_{i=0}^{3}b_{i}T^{-i};\;C=\sum_{i=1}^{4}c_{i}T^{-i}.$$

On the other hand, in this paper use is made of other published virial coefficients as well as those from this laboratory to provide a more extensive tabulation from about 20 to 423 °K to which are fitted expressions having forms suggested by the use of the Lennard-Jones potential. All coefficients were determined by the method of least squares.

## 2. Derivation of the Virial Coefficients

The virial expansion [5] may be rearranged to a form which, when truncated, is linear in density on isotherms, convenient for graphical or analytical determination of the second and third coefficients,

Φ≡(Pυ−RT)υ=RTB+RTC/υ+…. (1)

Parameters *RTB* and *BTC* of eq (1) as truncated have been determined on isotherms by least-squares using the reported compressibility data [1] and the orthobaric densities [6]. The number of datum pairs, *n*, on each isotherm is given in column (a) of table 1. It was found by trial that data for densities above 0.007 g mol/cm^3^ diverge from the linear behavior of eq (1). These data were omitted, with the result that a maximum of eight points per isotherm were applicable above 29 °K. (The above maximum density for validity of eq (1) with only two terms is less than half the critical density of 0.0156 g mol/cm^3^ [6].) Derived values of *B* and *C* are given in tables 2 and 3, respectively, at integral temperatures from 24 through 100 °K.

Equation (1) also was used for smoothing and interpolation of compressibility data to the highest densities by admitting as many terms as required by the experimental precision [1]. At temperatures below critical (32.98 °K), a single isotherm of eq (1) was used to represent both vapor and compressed liquid. The number of datum pairs, *n*′, and number of terms, *N*, in polynomial (1) are given in column (b) of table 1 which includes also the maximum density multiplied by 10^3^ for each isotherm. With increasing number of terms, the mean deviation decreases more at an odd number than at an even number of terms. Since coefficients of the higher-order terms of these divergent, alternating polynomials show no regular temperature-dependence, the expansions cannot be interpreted as virial equations [7]. Nevertheless, values of *B* and *C* obtained in this way are presented in appendix, table 1, to meet the commonplace question of the effect upon them of an increased number of terms in eq (1). It may be seen that values of *B* are essentially the same as in table 2. Values of *C*, while of similar magnitude to those in table 3, scatter badly.

Uncertainty in *B*, estimated from eq (1) for low densities, is

$$\delta B=[2Z-1+C/\upsilon^{2}]\;\delta \upsilon -\upsilon Z\delta T/T$$ (1a)

where *Z*≡*Pυ*/*RT*, or approximately

δB≈υ[δυ/υ−δT/T]. (1b)

**Figure 1:**
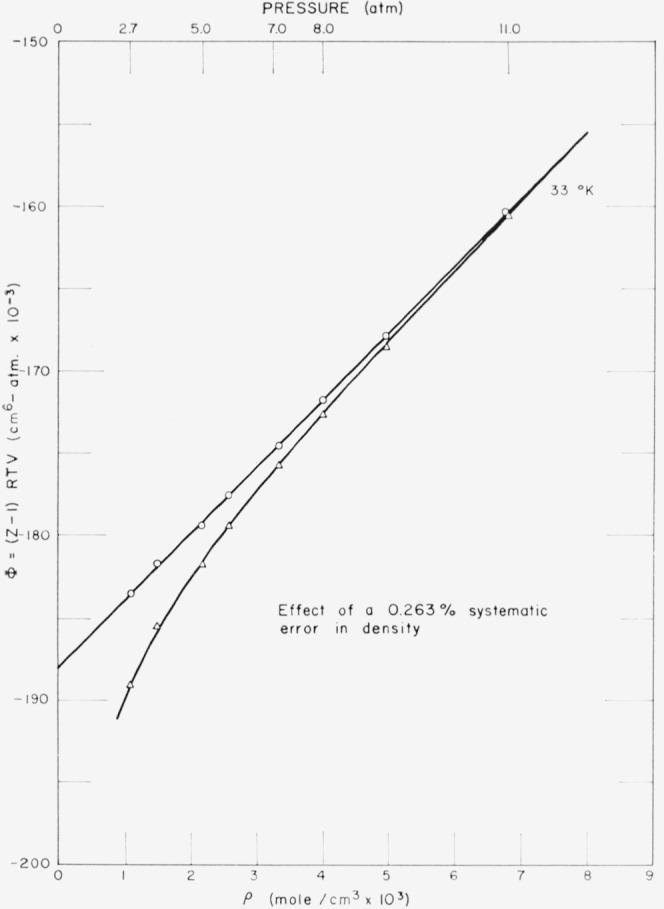
Effect of systematic error in density [2] upon the linear behavior of eq (1), for the 33 °K isotherm. The notation is *Z*≡*Pυ/RT.* Circles: experimental linear isotherm. Triangles: same data with artifically introduced error of 0.263 percent.

**Figure 2:**
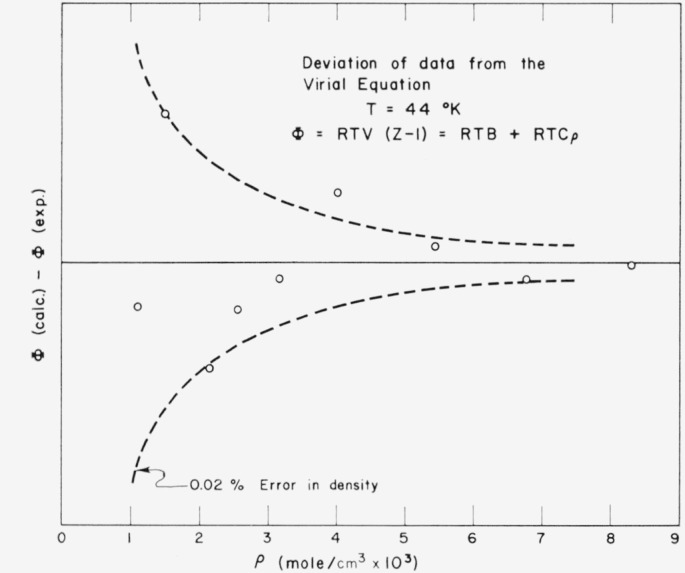
Deviations of data, *Φ≡ (Z−1)RTv*, where *Z ≡ Pv/RT*, from eq (1) at 44 °K. Dashed lines correspond to ±0.02 percent systematic error in density.

Experimental uncertainty in *υ* was estimated to be 0.2 percent at low densities [2]. Examination of the sensitivity of the shape of isotherms of (*Pυ*−*RT*)*υ* to errors in density, however, indicates a precision near 0.02 percent. This is illustrated by figure 1 in which the upper curve is an experimental linear isotherm, and the lower curve shows the effect of an artificially introduced error of 0.263 percent in density [2]. As a further illustration figure 2 gives deviations of the data from the least-squared, truncated virial expression at 44 °K and compares these with the boundary for a deviation of ±0.02 percent in density. From such considerations, the precision of these lowest experimental densities appears to be within 0.03 percent. Since this applies to all isotherms, the corresponding error in *B* should be roughly independent of temperature. For the lowest density, *υ*=1000 cm^3^/g mol; uncertainty in *B* thus is estimated to be about 0.3 cm^3^/g mol. Systematic deviations in the temperature-dependence of *B*, on the other hand, may be sought in absolute deviations of the NBS temperature scale for platinum resistance. For *δT*= 0.02 °K, *υ*=1000 cm^3^/g mol, and 25° ≤ *T* ≤ 100 °K, the range of uncertainty is 0.8≤*δB*≤0.2 cm^3^/g mol.

Since *B* is derived as the intercept of linear plots of (*Pυ/RT*−1)*υ* versus 1/*υ*, it may be seen that all of the above arguments for precision apply equally to absolute errors in *υ* or in *T.* Absolute uncertainty in *B* therefore is estimated to be in the range 1.1 cm^3^/g mol at 25 °K to 0.5 cm^3^/g mol at 100 °K.

To the above virial coefficients for parahydrogen from 24 to 100° in tables 2 and 3 have been added values for normal hydrogen at higher and lower temperatures in order to provide a more extensive set. Of the published values of virial coefficients for normal hydrogen [4, 8, 9, 10, 11, 12], values from Woolley, Scott, and Brickwedde [9] below 24 °K are included in table 2, while values from Michels, de Graaff, and Ten Seldam [10] at nonintegral temperatures above 98 °K are given in tables 2 and 3. Uncertainty of the data of Michels et al., was estimated by them to be 0.15 cm^3^/g mol in *B*, and about 15 percent in *C.* Excellent agreement of the independent data at 100 °K suggests that the virial coefficients of these hydrogen modifications may be indistinguishable at this and higher temperatures. Small differences have been detected at lower temperatures [13].

## 3. Representation of Second Virial Coefficient

**Figure 3:**
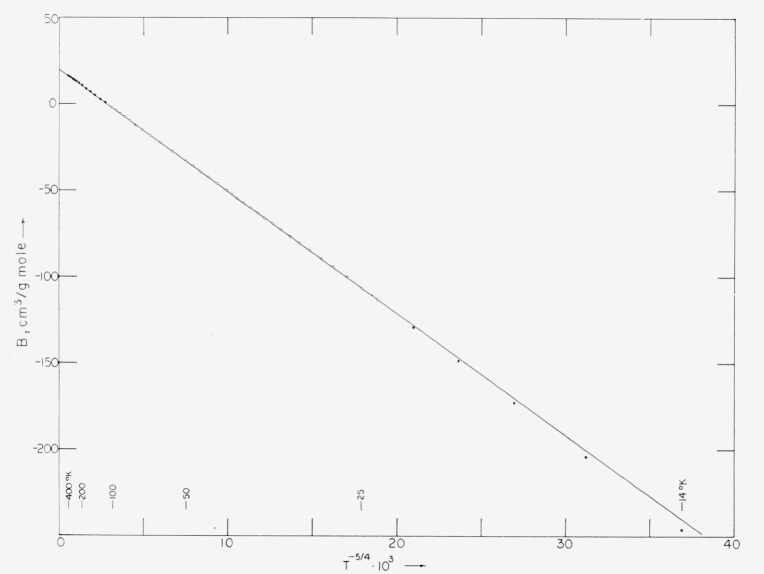
Second virial coefficient, *B*, versus *T*^−5/4^. Open circles are for parahydrogen. Filled circles for normal hydrogen below 21 °K are from [9] and above 100 °K from [10].

Figure 3 presents the derived data of table 2 as a function of *T*^−5/4^. The data for normal hydrogen at low temperatures are seen to deviate from the straight line extrapolated from the parahydrogen data, in agreement with the experimental comparison of the two modifications made by Beenakker et al. [13].

Systematic deviations persist with all analytical representations which have been investigated for the temperature-dependence of *B.* It therefore does not appear possible to select a form which is best for all purposes from among the following two, which utilize two and four constants, respectively, with notation *x* ≡ *T*_0_/*T:*

$$\begin{array}{ll} B=B_{0}[1-x^{5/4}], & T_{0}=109.83{}^{\circ}K,\\ B_{0}=19.866\;{\text{cm}}^{3}/\text{g mol}, & \Delta =0.125\;{\text{cm}}^{3}/\text{g mol}. \end{array}$$ (2a)

$$\begin{array}{ll} B=\sum_{i=1}^{4}B_{i}x^{(2i-1)/4}, & T_{0}=109.781{}^{\circ}K,\\ B_{1}=+42.464, & B_{2}=-37.1172,\\ B_{3}=-2.2982, & B_{4}=-3.0484,\\ \Delta =0.066\;{\text{cm}}^{3}/\text{g mol}. & \end{array}$$ (2b)

**Figure 4:**
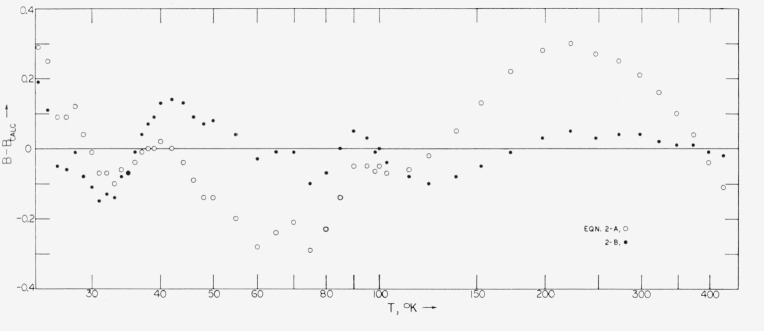
Deviations of second virial coefficient, *B*, in cm^3^/g mol, from eq (2a), open circles and from (2b), filled circles.

Mean deviations, given for each equation above, are defined as

$$\Delta \equiv {\left(n-N\right)}^{-1}\sum^{n}\left|B-B_{\text{calc}}\right|,$$

wherein *n*=49 is the number of datum pairs, and *N* is the number of constants in the equation. Calculated results from these equations are given in table 2, and the individual deviations are plotted in figure 4.

The form of (2a) is that used by Keesom for helium [14], the constant high-temperature limit corresponding to the rigid-sphere model for molecular interaction [5]. The value of *B*_0_ in (2a) is comparable with the volume 22.65 cm^3^/g mol of solid normal hydrogen at 4.2 °K [9]. The rather good fit provided by this simple equation is indicated by figure 3. Equation (2b) consists of the leading terms of an expansion derived from the Lennard-Jones potential [5]. Appendix, table II, gives coefficients for that expansion with increasing number of terms. (The form of the expansion given in the appendix does not include the root, *T*_0_.) It is seen that four terms give an acceptable representation. Whereas use of additional terms improves the fit, the similarity in form of the two sets of deviations in figure 4 suggests the presence of systematic experimental errors. If such errors are present, it is doubtful that use of higher terms would be justified. The form of the deviations in figure 4 having been found also with analytical representations of *B* other than polynomials, it is improbable that they arise from the selected polynomial forms of (2a) and (2b). It appears equally improbable that they arise entirely from deviations of the NBS temperature scale used.

## 4. Representation of Third Virial Coefficient

The Lennard-Jones potential yields an expansion in powers of *T*^−1/2^ [5]. From this key, eq (3) was evolved, using notation *x*≡*T*_0_/*T*,

$$\begin{array}{l} \;C=C_{0}x^{1/2}[1+cx^{3}][1-\exp(1-x^{-3})],\\ T_{0}=20.615{}^{\circ}K,\;C_{0}=1310.5{\left({\text{cm}}^{3}/\text{g mol}\right)}^{2},\\ \;c=2.1486,\;\Delta =17.4{\left({\text{cm}}^{3}/\text{g mol}\right)}^{2}. \end{array}$$ (3)

It represents the third virial coefficient within the apparent precision of the data. The last factor on the right of (3) differs significantly from unity only at temperatures below 40 °K. Calculated values of *C* are given in table 3.

The series

$$C=\sum_{i=1}^{n}C_{i}T^{-i/2}$$ (3a)

requires at least six terms to give a better representation than eq (3). The alternating series obtained by least-squares, however, are highly divergent; for example, the fifth term for *N*=6 exceeds the value of *C* by factor 240 at 24 °K.

## 5. Note on Two-Term Representations

The behavior of plots of *y*≡*B* or *C* as functions of *x*≡*T*_0_/*T* suggests an empirical relation of the form

$$y/y_{0}=x^{\mu }-x^{\nu }$$ (4)

with constants *μ*<*ν*. The limitations of (4) having been examined in obtaining (2) and (3) above, it is reasonable next to examine the relation

$$y/y_{0}=\exp\;(kx^{\mu })-\exp\;(kx^{\nu }).$$ (4a)

Since the five parameters in (4a) must be found tediously by trial, the exploration has been discontinued with preliminary results in the following table. It is concluded that (4a) may be sufficiently flexible to offer promise of accurate representations of both *B* and *C.*

$$\Delta \equiv {\left(n-3\right)}^{-1}\sum^{n}\left|y-y_{\text{calc}}\right|.$$

**Table:** 

*y*	*μ*	*ν*	*k*	*T*_0_	*y*_0_	*n*	Δ
*B*	¼	½	0.6	110.9	92.18	49	0.20
*C*	½	1	4	20	135	32	55

## Figures and Tables

**Table 1:** Number of datum pairs, n, and of terms N, for eq (1)

*T*, °*K*	(a)	(b)			*T*, °*K*	(a)	(b)		
	*n*	*n*′	*N*	10^3^/*υ*_min_		*n*	*n*′	*N*	10^3^/*υ*_min_
									
24	3	24	9	44.1	40	8	45	11	40.4
					42	8	42	11	40.3
25	3	25	9	44.1	44	8	41	9	39.7
26	4	25	9	43.8	46	8	40	9	39.1
27	5	27	9	43.8	48	8	39	9	38.4
28	6	29	9	43.4					
29	7	31	9	43.4	50	8	38	9	37.6
					55	8	37	9	36.6
30	8	33	11	42.9	60	8	36	9	35.9
31	8	35	13	42.9	65	8	34	9	34.6
32	8	38	13	42.5	70	8	33	7	34.0
33	8	47	15	42.4					
34	8	48	13	42.0	75	8	30	7	31.8
					80	8	29	7	30.8
35	8	48	11	42.0	85	8	28	7	29.8
36	8	47	11	41.5	90	8	27	7	28.9
37	8	47	11	41.5	95	8	26	5	28.1
38	8	46	11	40.9					
39	8	46	11	40.9	100	8	24	5	26.6

a For *N*= 2 and 0.001≤1/*υ*≤0.007 g mol/cm^3^.

b To the highest experimental densities, 10^3^/*υ*_min_.

**Table 2:** Derived and calculated values of B, cm^3^/g mol

*T*, °*K*	Derived	Calculated		*T*, °*K*	Derived	Calculated	
		(2a)	(2b)			(2a)	(2b)
							
15		−219.4	−222.3	50	−33.39	−33.25	−33.47
16	−204.2	−200.8	−202.8	55	−27.48	−27.29	−27.53
17		−184.7	−186.1	60	−22.70	−22.43	−22.67
18	−172.9	−170.6	−171.5	65	−18.64	−18.40	−18.63
19		−158.2	−158.7	70	−15.22	−15.01	−15.23
20	−148.8	−147.1	−147.4	75	−12.42	−12.13	−12.32
21		−137.2	−137.4	80	−9.88	−9.65	−9.82
22	−129.7	−128.4	−128.4	85	−7.63	−7.50	−7.63
23		−120.3	−120.3	90	−5.66	−5.61	−5.72
24	−112.8	−113.1	−113.0	95	−3.99	−3.95	−4.02
25	−106.2	−106.5	−106.3	98.15	−3.06	−2.99	−3.05
26	−100.3	−100.4	−100.3	100	−2.52	−2.47	−2.51
27	−94.80	−94.88	−94.74	103.15	−1.69	−1.62	−1.65
28	−89.66	−89.78	−89.65	113.15	+0.67	+0.73	+0.75
29	−85.03	−85.08	−84.96	123.15	2.63	2.65	2.73
30	−80.73	−80.72	−80.62	138.15	5.01	4.96	5.09
31	−76.75	−76.68	−76.60	153.15	6.89	6.76	6. 94
32	−72.99	−72.93	−72.86	173.15	8.84	8.62	8.85
33	−69.53	−69.43	−69.39	198.15	10.65	10.37	10.62
34	−66.22	−66.16	−66.14	223.15	11.98	11.68	11.93
35	−63.17	−63.09	−63.10	248.15	12.97	12.70	12.94
36	−60.26	−60.22	−60.25	273.15	13.76	13.51	13.72
37	−57.54	−57.53	−57.58	298.15	14.38	14.17	14. 34
38	−54.99	−54.99	−55.06	323.15	14.87	14.71	14.85
39	−52.60	−52.60	−52.69	348.15	15.27	15.17	15.25
40	−50.32	−50.34	−50.45	373.15	15.60	15.56	15.59
42	−46.19	−46.19	−46.33	398.15	15.86	15.90	15.87
44	−42.50	−42.46	−42.62	423.15	16.08	16.19	16.10
46	−39.18	−39.09	−39.28				
48	−36.17	−36.03	−36.24				

**Table 3:** Derived and calculated third virial coefficients

*T*,°*K*	*C.* (cm^3^/g mol)^2^		*T*,°*K*	*C*.(cm^3^/g mol)^2^	
	Derived	Calc’d.		Derived	Calc’d.
					
20	……	−405	50	964	968
21	……	+218	55	889	893
22	……	680	60	838	835
23	……	1018	65	785	789
24	1207	1259	70	743	750
25	1402	1425	75	726	718
26	1580	1533	80	694	690
27	1627	1596	85	659	665
28	1612	1625	90	636	643
29	1615	1629	95	624	624
30	1600	1614	98.15	530	613
31	1585	1586	100	609	606
32	1550	1549	103.15	560	596
33	1516	1507	113.15	540	567
34	1466	1463	123.15	560	542
35	1426	1418	138.15	540	510
36	1377	1373	153.15	522	483
37	1331	1330	173.15	500	454
38	1290	1290	198.15	458	424
39	1252	1251	223.15	437	399
40	1209	1215	248.15	415	378
42	1144	1151	273.15	404	360
44	1091	1095	298.15	370	345
46	1046	1047	323.15	340	331
48	1005	1005	348.15	313	319
			373.15	303	308
			398.15	310	298
			423.15	302	289

## Footnotes

1 Figures in brackets indicate the literature references at the end of this paper.

## 6. Appendix

**Table I:** Virial coefficients from extended isotherms of eq (1)

*T*, °*K*	*B*, cm^3^/g mol	*C*, cm^3^/g mol^2^	*T*, °*K*	*B*, cm^3^/g mol	*C*, cm^3^/g mol^2^
					
24	−115.3	5601	40	−50.18	1027
			42	−46.02	919
25	−106.5	1952	44	−42.54	1134
26	−101.1	2736	46	−39.25	1108
27	−95.01	2032	48	−36.28	1103
28	−90.36	2624			
29	−85.72	2561	50	−33.49	1050
			55	−27.67	1068
30	−80.96	1725	60	−22.93	1057
31	−77.24	2180	65	−18.77	906
32	−73.69	2642	70	−15.30	809
33	−69.14	554			
34	−66.27	1549	75	−12.57	854
			80	−9.96	767
35	−63.29	1537	85	−7.58	615
36	−60.30	1402	90	−5.57	559
37	−57.51	1280	95	−4.00	637
38	−55.04	1335			
39	−52.66	1321	100	−2.52	620

**Table II:** Polynomial coefficients in Lennard-Jones expansion for second virial coefficient $B=\sum_{i=1}^{N}b_{i}T^{-(2i-1)/4},({\text{cm}}^{3}/\text{g mol}).$(2b)

*N*	3	4	5	6
*b_i_*				
				
*b*_1_	+1.18579.10^2^	+1.37452.10^2^	+1.45098.10^2^	+1.20054.10^2^
*b*_2_	−7.32017.10^2^	−1.25884.10^3^	−1.55190.10^3^	−3.70405.10^2^
*b*_3_	−5.21897.10^3^	−8.16662.10^2^	+3.04443.10^3^	−1.78006.10^4^
*b*_4_		−1.13500.10^4^	−3.24136.10^4^	+1.41284.10^4^
*b*_5_			+4.07085.10^4^	−6.48921.10^3^
*b*_6_				+1.05121.10^6^
Δ	0.2991	0.0664	0.0615	0.0408
